# How to improve antibiotic awareness campaigns: findings of a WHO global survey

**DOI:** 10.1136/bmjgh-2018-001239

**Published:** 2019-05-09

**Authors:** Benedikt Huttner, Mirko Saam, Lorenzo Moja, Karen Mah, Marc Sprenger, Stephan Harbarth, Nicola Magrini

**Affiliations:** 1 Infection Control Program and Division of Infectious Diseases, World Health Organization Collaborating Centre on Patient Safety, Geneva University Hospitals and Faculty of Medicine, University of Geneva, Geneva, Switzerland; 2 World Health Organization, Department of Essential Medicines and Health Products, World Health Organization, Geneva, Switzerland; 3 Communications in Science, Geneva, Switzerland; 4 World Health Organization, Antimicrobial Resistance Secretariat, Geneva, Switzerland

**Keywords:** anti-bacterial agents, health communication, health education, drug resistance, bacterial, global health, World Health Organization, drug resistance, bacterial, awareness

## Abstract

**Introduction:**

We aimed to examine the characteristics of antibiotic awareness campaigns (AAC) conducted on a national or regional level since 2010.

**Methods:**

In October 2016, the WHO invited stakeholders involved in the planning or conduct of AACs to answer a web questionnaire. We solicited general information about the characteristics of the AAC, with a particular focus on key messages supporting optimal use of antibiotics.

**Results:**

Stakeholders in 93 countries were contacted and 55 countries responded. Overall, 60 AACs from 16 low/middle-income countries (LMIC) and 31 high-income countries were identified. Forty-five campaigns (75%) were conducted on a national level and most of them (47/60; 78%) were organised by public health authorities and publicly funded. There were no major differences between LMICs and high-income countries in the types of key messages. The scientifically questionable ‘Finish your prescription’ slogan was used by 31 AACs (52%). A One Health approach was mentioned in 13/60 AACs (22%). Most messages were universally applicable; adaptation to locally prevalent public misconceptions was not systematic. The evaluation of the impact of campaigns was still incomplete, as only 18 AACs (30%) assessed their impact on antibiotic use.

**Conclusion:**

For future AACs, it seems essential to base messages more rigorously on scientific evidence, context specificities and behavioural change theory. A new generation of messages that encourage first-choice use of narrow spectrum antibiotics is needed, reflecting international efforts to preserve broad spectrum antibiotic classes. Evaluation of the impact of AACs remains suboptimal.

**Key questions**

**What is already known?**

- Public knowledge on appropriate use of antibiotics tends to be low and antibiotic awareness campaigns (AAC) have been suggested as an intervention to improve outpatient antibiotic use. Recent, comprehensive information regarding the characteristics of AACs is lacking.

**What are the new findings?**

- Numerous countries have conducted AACs but public communication and key messages are not always supported by evidence, nor targeted to conditions for which inappropriate use is highly prevalent (eg, urinary tract infections). The evaluation of AACs remains suboptimal.

**What do the new findings imply?**

- Funding agencies should dedicate sufficient resources for the development and implementation and for the evaluation of AACs.
- Experts in health communication, social marketing and infectious diseases should be involved in the planning and conduct of AACs.
- AAC messages should be updated regularly reflecting local misconceptions and context.

## Introduction

Tackling antimicrobial resistance (AMR) is being increasingly recognised as a global priority and the WHO has set clear objectives to achieve this aim in its Global Action Plan on Antimicrobial Resistance.^1 2^ Antimicrobial use in humans is one of the key drivers of AMR,^3^ but yet it remains unclear what the most effective interventions are to improve antimicrobial use and reduce the spread of AMR.^4–6^ Communication to public on the association between unnecessary antimicrobial use and the emergence and spread of AMR seems an important component of strategies to control AMR.^7 8^ In fact, the vast majority of antimicrobials for human use are prescribed in the outpatient setting, often for upper respiratory tract infections where a benefit is either marginal or non-existent, since the majority of these infections are of viral origin.^9 10^ It has been repeatedly shown that patient knowledge, beliefs and attitudes may drive excessive antimicrobial use, either directly by influencing consultation seeking and self-medication by patients, or indirectly by influencing prescribing behaviour by physicians.^11^ A multicountry public awareness survey on AMR performed by WHO in 2015 highlighted that levels of public knowledge around the appropriate use of antibiotics vary among countries, but tend to be low across all WHO regions.^12^ Furthermore, physicians and pharmacists in many countries report feeling pressured by patients to prescribe or dispense antibiotics.^13^ It seems therefore logical to try to improve knowledge and modify beliefs and attitudes of the general public with regard to antibiotics through large-scale antibiotic awareness campaigns (AAC).^14 15^

Properly planned and conducted AACs should provide high-quality health communication to individuals. However, some messages on antibiotic use promoted in AACs have raised concerns. One message included in the materials supporting the 2016 World Antibiotic Awareness Week coordinated by WHO was, for example, criticised for not being evidence based and unlikely to produce relevant changes in AMR^16^: patients were advised to ‘always complete the full prescription, even if you [they] feel better, because stopping treatment early promotes the growth of drug-resistant bacteria.’^17^ While this is true for tuberculosis, some studies suggest that longer courses of therapy can result in more likely emergence and selection of AMR in other infections.^18 19^ Furthermore, if the rationale for conducting AACs is that a large proportion of prescriptions are unnecessary, it seems counterintuitive to actively encourage patients to finish superfluous treatments (in countries with endemic tuberculosis the situation may be different).

To examine whether and how countries had conducted AACs in the recent past, and which key messages were conveyed to the public, we conducted a review of practices, messages and outcomes of AACs across high, middle and low-income countries.

## Methods

In March 2016, the WHO Antibiotics Planning and Methods Working Group of the Model List of Essential Medicines (EML) recommended complementing the revision of the antibiotic section of the EML with a survey of large-scale AACs. In response to this call, the Secretariat of the EML formed a partnership with the WHO Collaborating Centre on Patient Safety at Geneva University Hospitals, which had previously conducted a systematic review of characteristics and effects of AACs.^20^

### Definitions

For the purpose of this survey, the term AAC was defined as a comprehensive effort to disseminate information about responsible use of antibiotics and the risks of antibiotic misuse to the lay public. We included campaigns that targeted healthcare professionals in addition to the lay public, but activities focusing mainly or solely on healthcare professionals were excluded. We also excluded campaigns focusing only on tuberculosis or antibiotic use in animals. We aimed to identify AACs conducted on an international, national or regional level, excluding those conducted in single communities or hospitals. Countries were classified in high, medium or low-income categories according to the July 2016 World Bank list of gross national income per capita.^21^ Additionally, countries were classified by WHO region.^22^

### Survey content

A pdf of the complete survey is available as online supplementary appendix 1. Briefly, topics covered by the survey included:

10.1136/bmjgh-2018-001239.supp1 Supplementary data

- Information about the survey respondent (eg, name, function, contact details).
- General information about the AAC (eg, national vs regional, name, years conducted, participation in World Antibiotic Awareness Week, organisation and funding, budget).
- Target audience and targeted infections.
- Key messages (respondents could select from a list of generic key messages and add free text if desired).
- Interventions implemented (eg, type of interventions, type of communication material).
- Evaluation of the campaign.

### Target respondents

Stakeholders to be contacted for this survey were identified through a preliminary review of the scientific literature and previously established contacts of the survey team. Representatives of national health ministries and non-governmental organisations (NGO) were contacted through WHO regional offices and national focal points.

### Survey tool

The survey was administered using SurveyMonkey (SurveyMonkey, San Mateo, California, USA).

### Analysis

All data were exported into an Excel spreadsheet (Microsoft, Redmond, USA), checked for accuracy and analysed in Excel for descriptive analysis.

## Results

**Figure 1:**
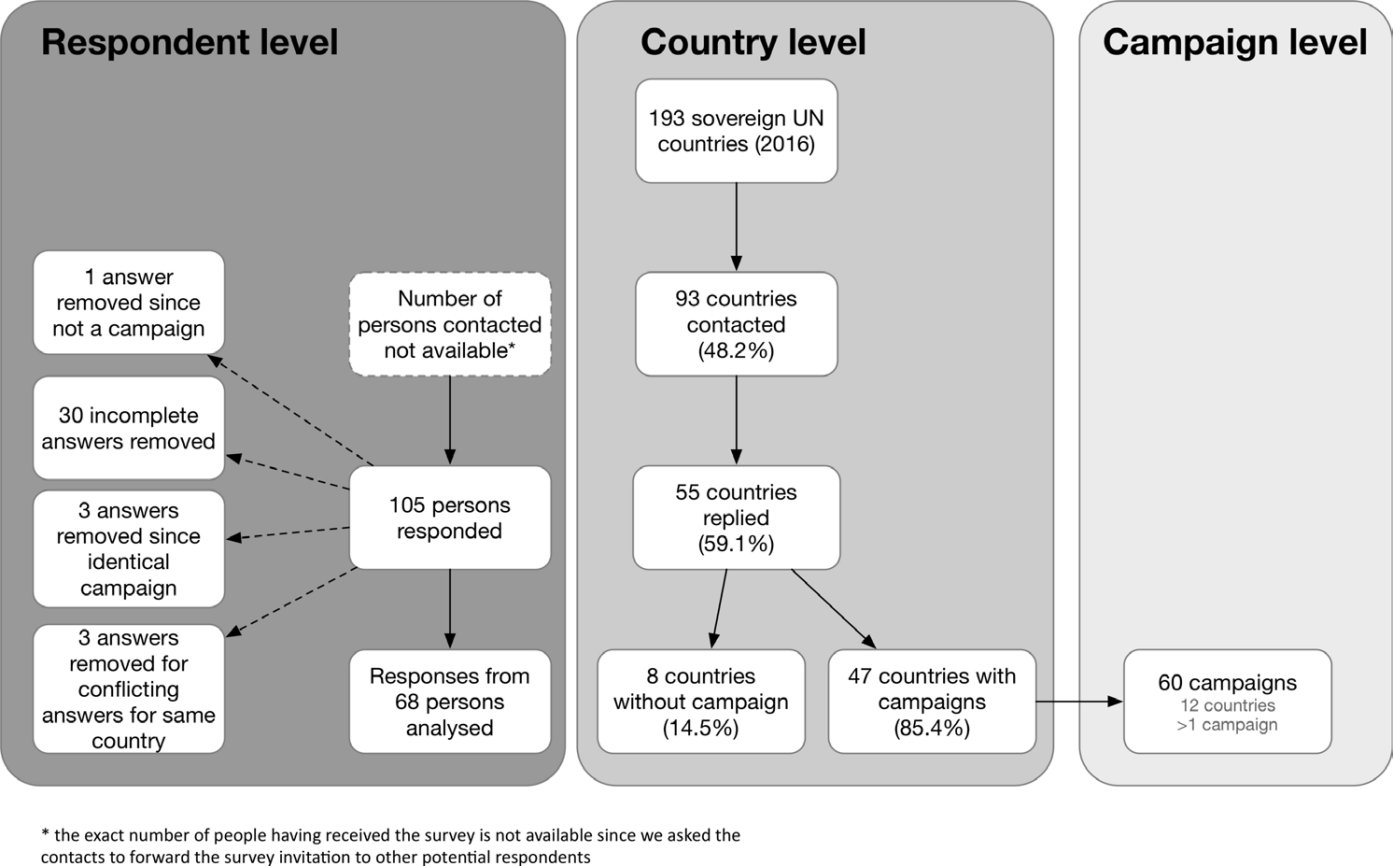
Survey flow chart. UN, United Nations.

In early October 2016, invitations to participate in the survey were sent to national health ministries, WHO country offices and representatives of NGO in 93 (48%) of the 193 sovereign states that were members of the United Nations in 2016 (in some countries more than one person was contacted) (figure 1). Reminders to participate were sent on October 25 and November 4 2016. We received answers from 105 persons from 56 countries (country-level response rate: 56/93; 60%). Follow-up emails were sent to 44 respondents who initially provided incomplete answers; eventually, 30 answers that remained incomplete despite the reminders were removed from the analysis. In the six instances when several answers were received for the same campaign, only those provided by the official coordinators of the campaigns were taken into account, since it was assumed that these answers would be more reliable. Conflicts among answers by different respondents regarding the existence of a campaign emerged in nine countries. In these cases, only the answers mentioning the existence of an AAC were taken into account.

### Geographic location of campaigns

**Figure 2:**
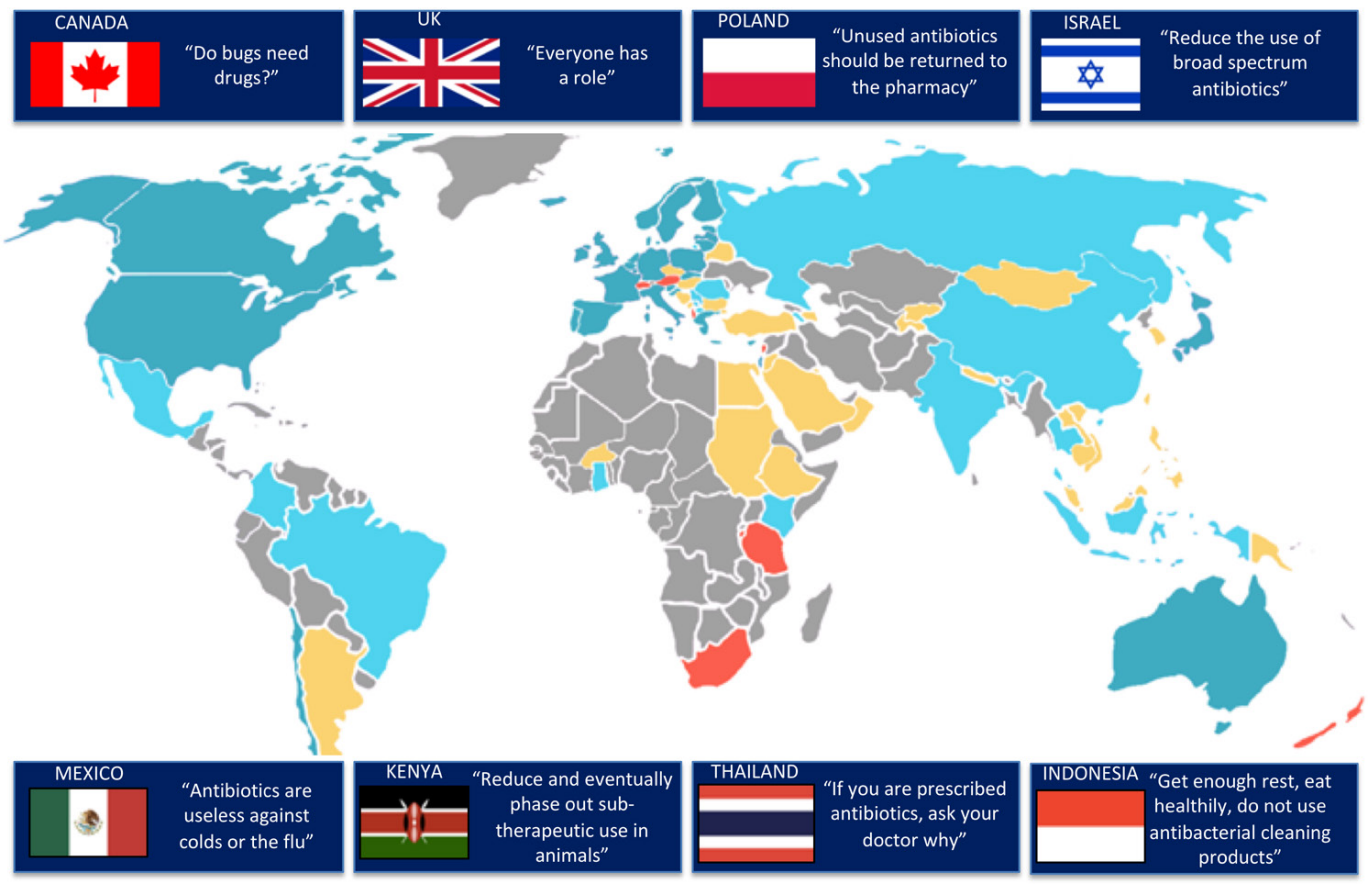
Map of countries included in the survey, with examples of key messages. Blue: antibiotic campaign conducted since 2010 (low/middle-income countries [LMIC]: light blue/high-income countries: dark blue). Red: answered ‘no campaign’ OR ‘not sure’. Yellow: contacted, but no answer received. Grey: not contacted.

**Figure 3:**
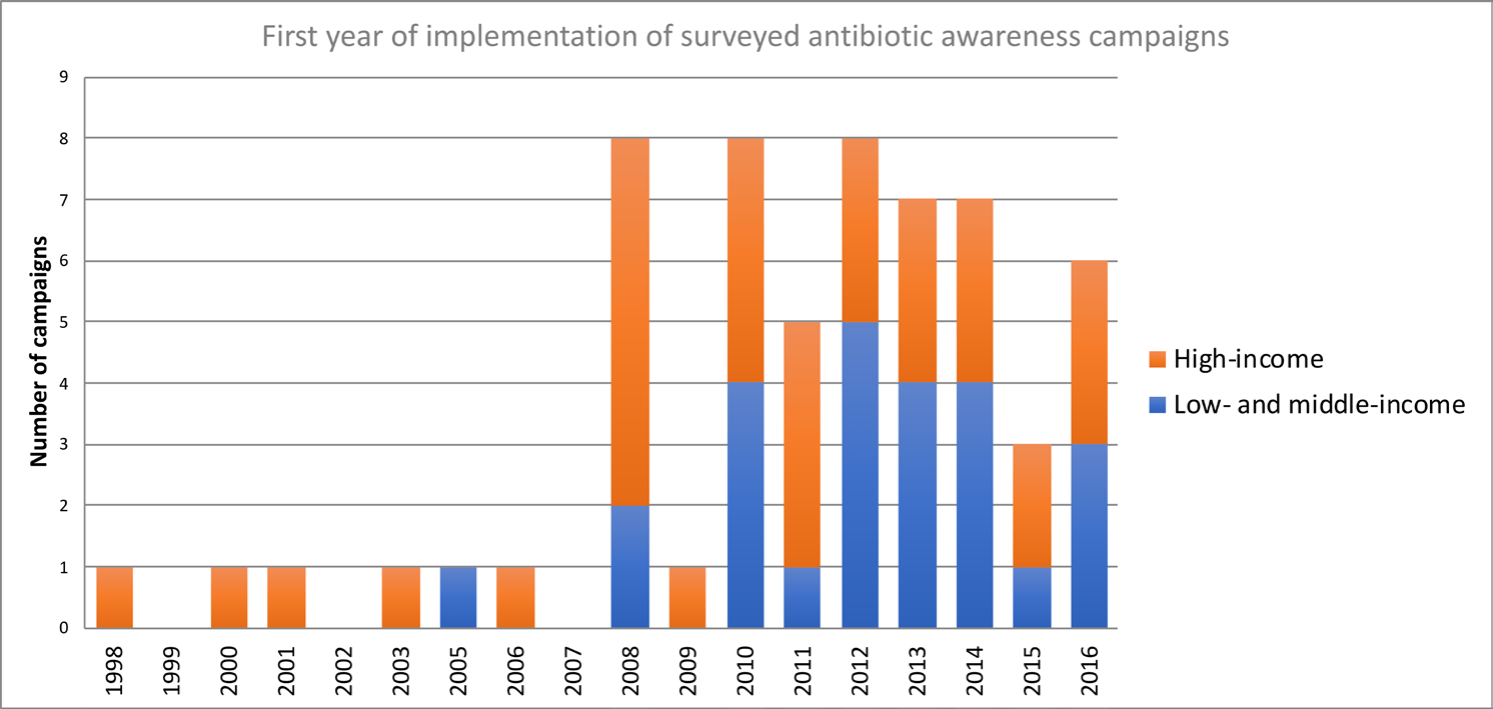
First year of campaign implementation.

Sixty campaigns were identified; of these 16 were conducted in low/middle-income countries (LMIC) and 31 high-income countries (12 countries ≥1 campaign) (figure 2). From eight countries we received answers stating the absence of campaigns since 2010; 38 (40%) countries failed to answer the survey, despite reminders. Categorised by WHO region, 35 (58%) were from the European region, 9 (15%) from the region of the Americas, 6 (10%) from the South-East Asia region, 5 (8%) from the Western Pacific region, 3 (5%) from the African Region and 2 (3%) from the Eastern Mediterranean region. The majority of campaigns (45/60, 75%) were implemented at the national level while 15 (25%) were implemented at regional level. Most AACs had been started relatively recently, with a notable increase in the number of campaigns since 2008, the year when the European Antibiotic Awareness Day was implemented for the first time (figure 3).^23^ About two-thirds (38/60; 63%) of AACs were still active at the time of the survey.

**Figure 5:**
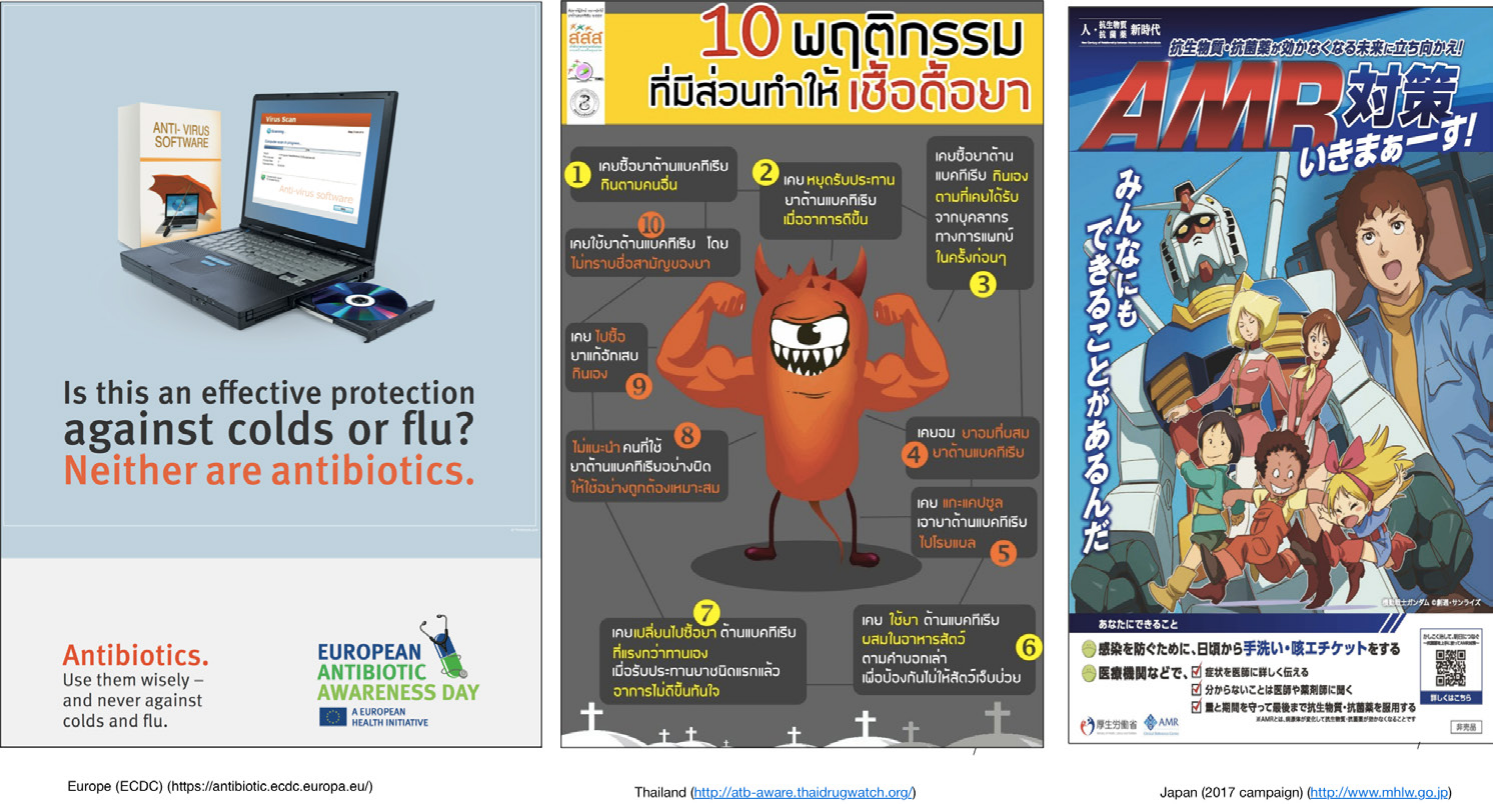
Examples of campaign material used in antibiotic awareness campaigns in Europe, Thailand and Japan.

### Target population and interventions

With the exception of 12 (20%) campaigns all targeted the general public and physicians simultaneously; the specifically targeted groups in the population (eg, caregivers, parents of little children) and among healthcare professionals (eg, paediatricians, general practitioners) varied, however, widely. Education and communication material was distributed in print (52/60, 87% of the campaigns), online (46/60, 77%) or both (42/60, 70%). Thirty-four campaigns (57%) used television and 28 campaigns (47%) used radio ads to reach the public. Further interventions comprised public relation activities (38/60, 63%), press conferences (32/60, 53%), educational meetings for prescribers (29/60, 48%) and active promotion of treatment guidelines (28/60, 47%). Most AACs (47/60; 78%) were organised by public health authorities and publicly funded.

### Key messages and target conditions

**Figure 4:**
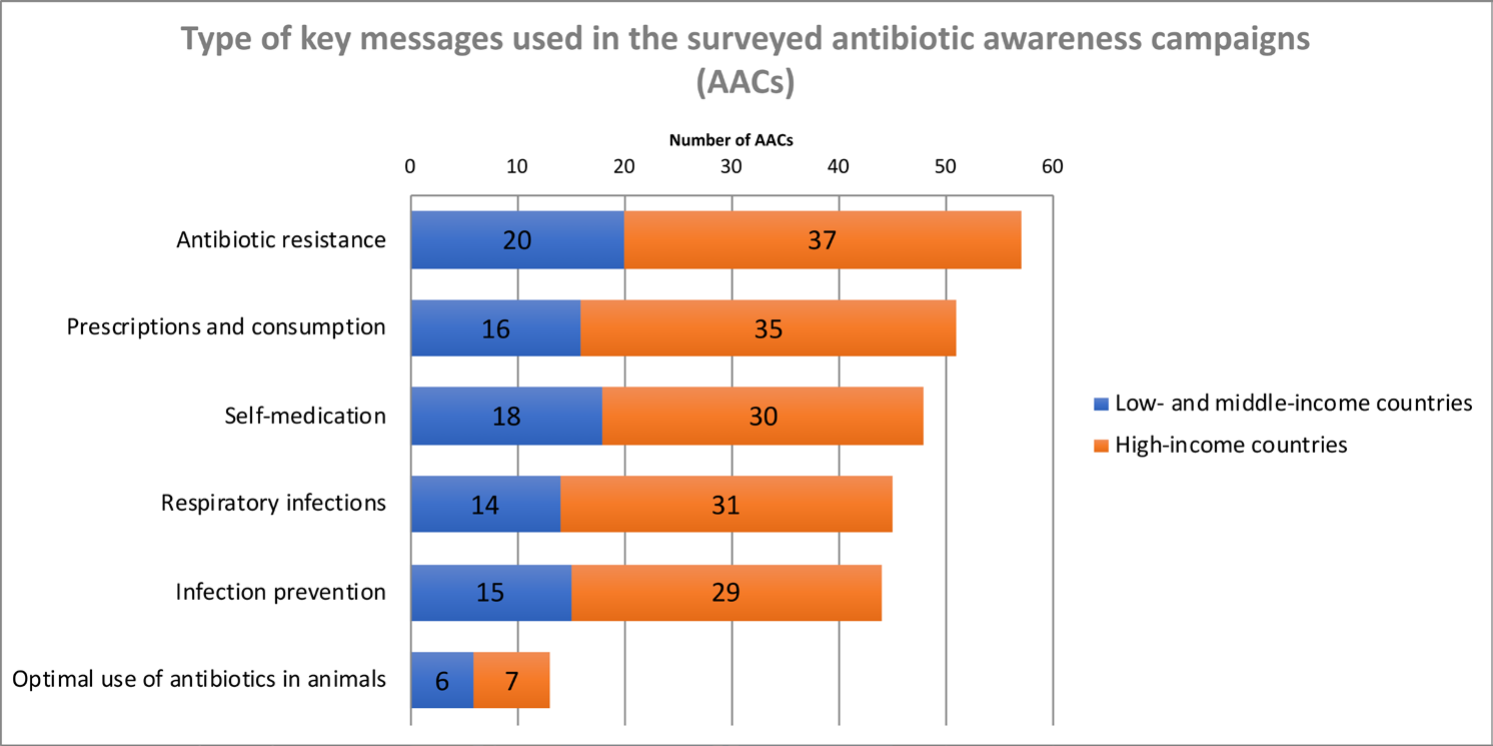
Topics covered by key messages in the campaigns.

The types of key messages used in the AACs were similar independent of the income status of the countries (figures 2, 4 and 5). Messages addressing AMR, such as: ‘Misuse and overuse of antibiotics cause resistance’ (47 campaigns, 78%); ‘If we use antibiotics incorrectly we will lose them/they will become ineffective’ (43, 72%); or ‘Antibiotic resistance is an important problem’ (40, 67%), were the most prevalent and used by all but three campaigns (95%). Other common slogans (used by 51 AACs, 85%) were related to the prescription and consumption of antibiotics, predominantly expressing the idea that ‘Judicious/Prudent/Responsible/Appropriate/Adequate use of antibiotics is important’ (41 campaigns, 68%). With regard to negative consequences of antibiotic use not directly related to AMR, a minority of campaigns (25, 42%) conveyed the message that antibiotics have side effects. Messages related to self-medication were used frequently (48, 80%), in particular the messages ‘Do not buy/use antibiotics without a prescription’ (26, 43%) or ‘Do not save leftover antibiotics/Discard leftover antibiotics’ (30, 50%). This survey failed to identify messages that specifically advocated reductions in the length of antibiotic courses; on the other hand, about half of AACs (31, 52%) used some form of the ‘Follow/Finish the antibiotic prescription (in dosage and duration)’ message in their campaign material.

Respiratory tract infections other than influenza were the most frequently targeted condition (46, 77%); 36 (60%) campaigns also specifically addressed influenza (figure 4). Other types of infections were less frequently addressed: urinary tract infections and sexually transmitted diseases were targeted by 15 (25%) and 6 (10%) campaigns, respectively; 3 (5%) campaigns also addressed diarrhoea/gastrointestinal infections, and two skin infections. Messages related to infection prevention and control were incorporated by 75% (45) of campaigns, most often (41, 68%) focusing on the importance of hand washing to limit the spread of pathogens. Messages addressing vaccination were part of 24 campaigns (40%).

A ‘One Health’ approach, incorporating messages mentioning the use of antibiotics in animals, was adopted by 13 (22%) campaigns, with nine campaigns (15%) conveying the message ‘Don't give antibiotics to your pet; consult a veterinarian first.’ Six campaigns (10%) simultaneously targeted pet owners and veterinarians. In general, AACs were tailored either to humans (ie, patients and prescribers), or to the animal sector (ie, farmers and veterinarians) with different government agencies responsible for the respective implementation (eg, ministries of health, agriculture, and so on).

### Evaluation of campaigns

Twenty-five campaigns (42%) have been formally evaluated: 18 (30%) campaigns evaluated the impact on antibiotic use, 13 (22%) monitored the impact on AMR, 11 (18%) assessed recall, 16 (27%) assessed knowledge of the public and 3 (5%) looked at consultation behaviour. For most campaigns the results of these evaluations are, however, not publicly available.

### Barriers and recommendations

Respondents for 24 AACs (40%) answered the question regarding whether they would recommend AACs to other countries based on their experience: all but four, who were ‘not sure’, would do so. The most frequently mentioned barriers to implementation of AACs were the lack of funding (mentioned by 11/24 [46%]) and lack of political support (6/24; 25%). Other perceived barriers mentioned were the difficulty to explain a complicated topic (ie, AMR) to people with limited educational background, lack of guidelines, resistance and inertia of healthcare professionals and the difficulty to coordinate a campaign across multiple sectors.

## Discussion

In this international survey, we were able to identify 60 AACs conducted in 47 countries since 2010. A wide variety of key messages were used in these campaigns, but not all of them seem scientifically sound (see below). The lack of thorough evaluation, the absence of prospectively determined control groups and the multifaceted nature of most campaigns represent major obstacles to make any inferences about the overall effectiveness of the campaigns, let alone which interventions and key messages work best. These results, which now include a limited number of LMICs, reinforce the results of our previous review.^20^ Of note, our survey did not show major differences between AACs in LMICs and high-income countries, except them being more frequent in the latter.^20^ Despite the fact that countries do have different levels of antibiotic (mis)use, access to antibiotics, AMR and incidence of infectious diseases, the standardisation and synchronisation of arguments among AACs prevails.^24^ In our review of AACs in high-income countries conducted between 1990 and 2007,^20^ we found no mention of the One Health concept, whereas this should be more represented since the problem of AMR is not limited to human health. The advantage associated with uniformity of practices and messages across countries is ambiguous, as it shows consistent global messages but limited adaptation to local situations. As it seems strategic to maintain AAC as a priority for countries to develop a common background knowledge for optimal antibiotic use, there is a need to integrate global strategies with better communication techniques to meet specific local knowledge gaps and misuses, while protecting access to antibiotics for those who need them.

### Key messages

Though most key messages used in AACs seem scientifically sound, a few could require a review or some comments. The validity and usefulness of the ‘Complete the course’ message has, for instance, been questioned, since antibiotics are often prescribed unnecessarily and for too long, and the evidence for the impact of duration of treatment on AMR is weak.^16^ Given these concerns, WHO has eliminated since 2017 this message from World Antibiotic Awareness Week. (box 1 illustrates some achievements and challenges faced by this initiative).^14^

Box 1 **World Antibiotic Awareness Week (WAAW): a global platform**

The first strategic objective listed in the Global Action Plan on Antimicrobial Resistance (AMR) is to improve awareness and understanding of AMR through effective communication.

**Current communication strategy and its results**

WHO has implemented an annual global awareness campaign called ‘Antibiotics: Handle with Care,’ which was first launched in November 2015 during the first WAAW. A total of 131 countries officially reported WAAW activities in 2017 in all six WHO regions, suggesting that a remarkable amount of energy was invested in AMR communication. However, this amount of energy resulted in a very heterogeneous scenario, with some countries providing the public, policymakers and health professionals with basic passive background information and others delivering actionable messages and building a consensual agenda with professional and civil society to improve antibiotic use. These national and international awareness stimuli lend themselves to the development of a global attitude shift towards antibiotic preservation and AMR containment. In 2017, there was unprecedented social media interaction focused on tripartite (ie, concerning humans, animals and the environment) AMR materials developed by WHO with the Food and Agriculture Organization (FAO) of the United Nations and the World Organisation for Animal Health (OIE). There were more than 15 million user interactions for WHO material regarding this issue on three social media platforms (Facebook, Twitter, Instagram).

**Challenges ahead**

The WHO attempt to harmonise the communication on AMR among its six regions is challenged by disparities in awareness, beliefs and attitudes about antibiotics. Different education strategies are required to reduce disparities, yet we have little evidence regarding the success of a range of interventions in reducing these inequities. Effective health communication science must be agile, revising messages that become obsolete (eg, finish your course guidance) and revising delivery strategies (eg, use of local dialects, message framing, media). The next generation of WAAW messages should be developed through user testing process with stakeholder groups at various levels, across different regions for relevance and contextual accuracy. Motivational consequences of narratives, including offence and unintended consequences, should be part of communication risk management. Messages should target both individual and contextual factors showing the interplay of responsibilities between single and social actions or dispositions. Finally, there is a concrete need to refine awareness indicators to be included as part of national action plan implementation on AMR control.

Not to skip antibiotic doses might be a message with a more direct link to AMR, although it seems challenging how to best convey such subtle differences to the public. For tuberculosis, the message to ‘Complete the course’ is obviously valid and in countries where this disease is endemic, there may be a role for this message in AACs. The message ‘Do not save leftover antibiotics/Discard leftover antibiotics’ occurred frequently. In settings where access to antibiotics is limited, this message might clash with the prevailing economic reality. The use of messages perceived as irrational or unrealistic might diminish the credibility of AACs; messages should thus be carefully evaluated and crafted to reflect local conditions.

In the USA, antibiotics are the most common cause of emergency department visits for adverse drug events in children <18 years; furthermore, the negative impact on the human microbiome starts to be increasingly recognised.^25 26^ Nevertheless, only relatively few campaigns conveyed messages about the potential direct negative impact of antibiotic use on the patients. We acknowledge, however, that this is a balancing act since potential unintended consequences, such as patients refusing antibiotics when they would be actually indicated, cannot be excluded. In this context, it is worth mentioning that the issue whether campaign messages should be preferentially framed as ‘positive’ (eg, ‘The appropriate use of antibiotics keeps them effective’) or ‘negative’ (eg, ‘Inappropriate use of antibiotics makes them ineffective’) remains unresolved.^27 28^ AMR in bacteria causing sexually transmitted diseases (such as gonorrhoea) and urinary tract infections is on the rise worldwide.^29–31^ Few AACs targeted these infections and it may be relevant to expand the scope of future campaigns to cover them and evaluate the impact.

Some messages seem more suited for countries without access problems or where self-medication is common. It seems vital that AACs address locally prevailing public misconceptions and adapt messages to the local culture, especially since there is some evidence that (admittedly hard to quantify) cultural differences among populations are associated with different behaviours related to antibiotics.^32–36^ The messages ‘Do not buy/use antibiotics without a prescription’ appeared equally in countries with or without apparent problems related to antibiotic access without prescriptions. On the other hand, in Russia, Serbia, China and India, where between 57% and 67% of the population believe that ‘It’s okay to use antibiotics that were given to a friend or family member, as long as they were used to treat the same illness,’^20^ none of the campaigns used the slogan ‘Do not share antibiotics’ (or a variation of it). Overall, there seems to be a need to better explore cultural difference in attitudes towards antibiotics, for example, through interviews and focus groups, so that AACs can better tailor their messages to specific misconceptions within a country.^37 38^

We feel that messages related to vaccination, particularly vaccines against influenza and pneumococci, could be used more frequently in AACs. Many patients with influenza-like illness unnecessarily receive antibiotics, which could be partly prevented through use of the influenza vaccine.^39 40^ Furthermore, the inclusion of the pneumococcal conjugate vaccine in routine childhood immunisation schedules in many countries has been associated with a decrease in infections caused by penicillin-non-susceptible pneumococci, because the strains included in the vaccine tend to be more resistant to antibiotics.^41 42^

A common message of AACs is that ‘Antibiotics do not kill viruses/are not effective against viruses.’ It is, however, not clear whether the attempt to convey this fact about the biology of infections is effective. In Europe, the 2016 Eurobarometer survey on AMR showed that in France and Belgium, two countries with a long history of mass media AACs, the question whether ‘Antibiotics kill viruses’ is now answered more often correctly by respondents than the European average (59% and 54% vs 43%), yet despite a reduction in outpatient antibiotic use associated with the AACs, it remains well above the European average in both countries.^43–45^ On the other hand, an Italian study found that the percentage of the surveyed population correctly answering the question whether antibiotics are active against viruses actually decreased after an AAC.^46^ Campaign messages had specifically addressed this issue, illustrating how difficult it is to convey even basic facts.

According to survey respondents, only 4 of the 60 (7%) campaigns involved psychologists or sociologists in their design or implementation. This seems disappointingly low, as it is increasingly recognised that an integrated social and communication science approach is necessary to improve the acceptability of campaigns focusing on behaviour change, as done in other areas of public health such as smoking cessation or HIV prevention.^47 48^ Targeting specific subgroups of the public and healthcare professionals, with tailored messages in a ‘person-centred approach’, seems worth investigating as a way to increase the effectiveness of AACs.^49^ Not even a third of the surveyed campaigns focused on school-age children and adolescents: embedding knowledge about antibiotics and AMR in school curricula could also be considered as an additional strategy for long-term behaviour change.^50 51^ Medical and veterinary schools should also be considered targets for awareness-raising activities. Finally, the use of social media in AACs merits further attention.^52^

### Impact of AACs on antibiotic use and other outcomes

Examples from Belgium and France have shown that national campaigns may reduce overall antibiotic consumption, but have difficulty sustaining these successes over time.^53 54^ Experiences from other public health campaigns show that repeated exposure of the targeted public over long periods of time is often necessary to exert a sustained effect.^47 55^ The lack of comprehensive (or even basic) evaluation of most AACs remains a key obstacle for the generation of a better evidence base. Very few AACs have been described and assessed in the peer-reviewed literature.^51 56^ A review estimated the impact of various public campaigns in Europe between 1997 and 2007 to be equivalent to a reduction of 1.3–5.6 defined daily doses per 1000 inhabitants per year in overall antibiotic use, but these data have to be interpreted with caution.^20 57^ Implementing an AAC should be associated with reasonable expectations. Nevertheless, several factors leading to increased success of AACs have been suggested, including carefully designed and simple key messages; targeting a wide audience such as patients, their families and healthcare workers; engaging physicians and other healthcare professionals early in the campaign and designing the key messages with them; using mass and social media; and continuously repeating key messages over long periods of time.^46 58^

AACs should include an assessment of many different indicators, including the impact of the campaign on patient knowledge, belief and attitudes to consultation behaviour, the quality of antibiotic prescribing and the potential undesired effects such as the number of reconsultations or hospitalisations for complications. The key obstacle to better evaluation seems to be the lack of funding for this purpose; funding agencies should consider this as a priority.

### Limitations of the survey

In the context of global health with increased political attention to AMR, our specific intent was to provide a snapshot assessment of recently implemented AACs. While we tried to be comprehensive, we were not able to obtain answers from all countries, with some WHO regions (such as Africa) clearly being under-represented. A response bias is also likely, with countries having conducted campaigns being probably more likely to answer the survey. Furthermore, due to time and resource constraints we provided the survey only in English. For most campaigns, the information regarding the AAC relied on the answers from a single respondent, making the provided data subject to recall bias and increasing the probability that an AAC may have been missed if the respondent was not aware of it. We also did not attempt to independently verify the answers. Finally, the questions for the survey were not formally validated, since they were based mostly on a previous inventory of AACs.

## Conclusion

AACs are widely used across the globe, yet many questions regarding how best to conduct and evaluate these campaigns remain unanswered. AACs should move beyond long-standing but problematic messages (eg, ‘complete the course’), towards accurate and locally adapted communication. Involvement of experts in health communication and social marketing seems crucial. Policymakers have to recognise the importance of evaluating them. Scientifically derived evidence must guide their design, implementation and evaluation by national governments, multilateral institutions and civil society organisations. The 2017 revision of antibiotics included in the EML categorised antibiotics into three groups (Access, Watch and Reserve [AWARE]), with the goals of improved access and clinical outcomes, reduced potential for development of AMR and preserved effectiveness of the so-called last-resort antibiotics.^59^ The next generation of AACs should consider how to integrate the new AWARE categorisation of antibiotics in public communication.
