# Supplementary material for: How to improve antibiotic awareness campaigns: findings of a WHO global survey
Source: BMJ Glob Health. 2019 May 9;4(3):e001239. doi: 10.1136/bmjgh-2018-001239 (PMC6528771; doi:10.1136/bmjgh-2018-001239)
Supplement: Supplementary data [file bmjgh-2018-001239supp001.pdf]

Evaluation of antibiotic awareness campaigns

Welcome to this survey!

Geneva University Hospitals and the World Health Organization are conducting this survey to collect information about the characteristics of - and obstacles encountered by - antibiotic awareness campaigns conducted around the world.

According to our records, you may have been involved in designing or coordinating an antibiotic awareness campaign.

Please answer the survey in your personal capacity, even if you were involved in the campaign on behalf of your government.

Answering this survey should take 15 to 30 minutes.

For the purpose of this survey, the term "antibiotic awareness campaign" is intended as a comprehensive effort to disseminate information about responsible use of antibiotics and the risks of antibiotic misuse to the lay public. For this purpose an "antibiotic awareness campaign" typically uses posters, pamphlets, billboards, radio and television ads, social media, etc. Health care professionals may also be targeted as part of the intervention, but interventions focusing mainly on health care professionals are beyond the scope of this survey.

If there is any written report available about the campaign evaluation we would be grateful if you were willing to share it with us. In addition we would appreciate if you could send any material

used in the campaign to benedikt.huttner@hcuge.ch and m.saam@inscience.ch.

Evaluation of antibiotic awareness campaigns

Information about the survey respondent

* Your name

* Your function in the context of the campaign

* Your country

* Your email address and/or phone number

* Has any antibiotic awareness campaign (as defined above) been implemented in your country since 2010?

- Yes
- No
- Not sure

Evaluation of antibiotic awareness campaigns

Questions for respondents from countries not having implemented campaigns since 2010

* How important are the following problems related to antibiotic use**in your country**?

	(1) Not at all important	(2)	(3)	(4)	(5) Very important
Limited access to antibiotics	◯	◯	◯	◯	◯
Inappropriate prescribing of antibiotics by physicians	◯	◯	◯	◯	◯
Antibiotic resistance	◯	◯	◯	◯	◯
Self-medication with antibiotics	◯	◯	◯	◯	◯

* Was the problem of widespread antibiotic use or antibiotic resistance addressed by policy actions at any time point in the past **in your country** ?

◯ Yes

◯ No

◯ Not sure

Evaluation of antibiotic awareness campaigns

Which policy actions were implemented?

☐ Restrict sale of antibiotics without a prescription

☐ Mandate antimicrobial stewardship programs in hospitals

☐ Provide education and training sessions for health care professionals

☐ Establish surveillance of antibiotic use and resistance

☐ Restrict use of antimicrobials in animal husbandry and agriculture

☐ Other (please specify)

Did any of these policy actions fail?

◯ Yes

◯ No

◯ Not sure

Evaluation of antibiotic awareness campaigns

If yes, which policy action(s) failed?

[ ]

Why do you judge the policy / policies a failure (failure meaning*e.g.* a lack of impact on antibiotic use)?

- [ ] Lack of adequate funding
- [ ] Lack of coordination among key stakeholders
- [ ] Lack of political support
- [ ] Scarce recognition of the relevance of the problem / competing priorities

Other (please specify)

[ ]

* Is your country considering to launch an antibiotic awareness campaign for the public?

- ( ) Yes
- ( ) No
- ( ) Not sure

Evaluation of antibiotic awareness campaigns

General information about the public awareness campaign in your country

* What is / was the geographic scope of the campaign?

- ( ) National
- ( ) Regional (e.g. a state / province / county / district or other sub-national administrative region)
- ( ) Other (please specify)

[ ]

Evaluation of antibiotic awareness campaigns

Name of region / district (if applicable)

* Campaign name in original language (if several names, most current)

* Campaign name in English (if several names, most current)

If available, please provide the URL of the campaign

* Is the campaign temporary, seasonal or active all year?

- [ ] Temporary (e.g. for a few weeks during the year but independent of the season)
- [ ] Seasonal (*e.g.* during winter)
- [ ] All year

* In which year was the campaign conducted for the first time?

* In which year was the campaign conducted for the last time?

If the campaign has not been conducted each year, please indicate how many times (seasons if seasonal, years if year round) the campaign has been conducted.

- 1
- 2
- 3
- 4
- 5
- 6 or more

* Is the campaign ongoing (if seasonal, planned for the next season)?

- Yes
- No
- Not sure

* Is your country participating in World Antibiotic Awareness Week?

- Yes
- No
- Not sure

* Is your country participating in European Antibiotic Awareness Day?

- Yes
- No
- Not sure

* Does your country have a national strategy (i.e. comprehensive effort that includes multiple components (messaging, grassroots outreach, media relations, government affairs, budget, etc.) to disseminate information to the public to address the problem of widespread antibiotic use or antibiotic resistance?

- Yes
- No
- Not sure

Evaluation of antibiotic awareness campaigns

Please briefly describe the strategy in relation to public awareness about responsible use of antibiotics

If available, please provide the URL where the national strategy / action plan can be accessed

* Who is providing funding for the campaign?

- [ ] Government (Ministry of Health etc.)
- [ ] United Nations Agencies
- [ ] Foundation(s) (charities and non-governmental organizations (NGOs))
- [ ] Public health insurance
- [ ] Private health insurance company / companies
- [ ] Pharmaceutical industry
- [ ] Professional society / societies
- [ ] Other (please specify)

What was the approximate funding per year for the campaign? (please also indicate the currency)

Evaluation of antibiotic awareness campaigns

Target audience and targeted infections

* Which categories of the public (i.e. not healthcare workers or policy makers) were targeted by the campaign?

- [ ] General public
- [ ] Parents of young children
- [ ] Caregivers / teachers of children
- [ ] School age children and adolescents
- [ ] The elderly
- [ ] Travelers
- [ ] Journalists
- [ ] Hospital patients
- [ ] Other (please specify)

* Were health professionals specifically targeted by the campaign?

- [ ] Yes
- [ ] No

Evaluation of antibiotic awareness campaigns

Which health professionals were targeted by the campaign?

- [ ] General practitioners / Family physicians
- [ ] Paediatricians
- [ ] Medical specialists (e.g. ENT doctors, pulmonologists etc.)
- [ ] Dentists
- [ ] Nurses / nurse practitioners
- [ ] Pharmacists
- [ ] Other (please specify)

Were any of the following types of infections targeted by the campaign?

- [ ] Respiratory tract infections (other than flu)
- [ ] Flu (influenza)
- [ ] Urinary tract infections
- [ ] Sexually transmitted diseases
- [ ] Other (please specify)

* Were any of the following involved in designing / conducting the campaign?

- [ ] Infectious diseases specialists
- [ ] Microbiologists
- [ ] Psychologists
- [ ] Professional societies
- [ ] International experts
- [ ] Advertisement companies
- [ ] Primary care physicians
- [ ] Patients / patient interest groups
- [ ] None
- [ ] Other (please specify)

Evaluation of antibiotic awareness campaigns

Key messages and essential public slogans of the campaign

* Did the campaign use any key messages relating to antibiotic resistance?

- ( ) Yes
- ( ) No

Evaluation of antibiotic awareness campaigns

Select the type(s) of key message(s) relating to antibiotic resistance here below.*Of note, we are mostly interested in the main content of the message, the exact phrasing can be different.*

- [ ] "Antibiotic resistance is an important problem"
- [ ] "Misuse/overuse of antibiotics causes resistance"
- [ ] "Antibiotic resistant bacteria can kill / are dangerous"
- [ ] "Antibiotics are losing their power / become ineffective"
- [ ] "Antibiotic resistant bacteria can spread to other people / are transmitted"
- [ ] "If we use antibiotics incorrectly we will lose them / they will become ineffective"
- [ ] "With antibiotic resistance, infections become untreatable / antibiotics lose their power"
- [ ] "Fighting antibiotic resistance is a collective responsibility / concerns all of us"
- [ ] "Antibiotic resistance is increasing / becoming more common"
- [ ] Other (please specify)

* Did the campaign use any key messages relating to prescriptions and consumption?

- ( ) Yes
- ( ) No

Evaluation of antibiotic awareness campaigns

Select the type(s) of key message(s) relating to prescriptions and consumption here below.*Of note, we are mostly interested in the main content of the message, the exact phrasing can be different.*

- [ ] "Judicious / prudent / responsible / appropriate / adequate use of antibiotics is important"
- [ ] "Antibiotics have side effects / adverse effects"
- [ ] "Antibiotics are not candy / antibiotics should be used responsibly"
- [ ] "Ask your doctor / pharmacist / other healthcare worker for advice on how to use antibiotics"
- [ ] "Follow / Finish the antibiotic prescription (in dosage and duration)"
- [ ] "Do not pressure healthcare professionals to prescribe an antibiotic"
- [ ] "Don't rush to your doctor when first symptoms appear: wait and see how your cold/ailment evolves"
- [ ] Other (please specify)

* Did the campaign use any key messages relating to self-medication?

- ( ) Yes
- ( ) No

Evaluation of antibiotic awareness campaigns

Select the type(s) of key message(s) relating to self-medication here below.*Of note, we are mostly interested in the main content of the message, the exact phrasing can be different.*

- [ ] "Take antibiotics only when you need them / when they are indicated"
- [ ] "Avoid self-medication with antibiotics"
- [ ] "Do not buy / use antibiotics without a prescription"
- [ ] "Discard leftover antibiotics / do not save leftover antibiotics"
- [ ] "Do not share antibiotics with others"
- [ ] Other (please specify)

* Did you use any key messages specifically relating to respiratory infections?

- ◯ Yes
- ◯ No

Evaluation of antibiotic awareness campaigns

Select the type(s) of key message(s) relating to respiratory infections here below.*Of note, we are mostly interested in the main content of the message, the exact phrasing can be different.*

- ☐ "Antibiotics do not work against colds / flu"
- ☐ "Antibiotics do not work against viruses"
- ☐ "Antibiotics only work against bacteria"
- ☐ "Most respiratory infections are of viral origin"
- ☐ "In case of cold / flu try treating symptoms first"
- ☐ "In most cases, a sore throat will last X days"
- ☐ Other (please specify)

* Did you use any key messages relating to infection prevention?

- ◯ Yes
- ◯ No

Evaluation of antibiotic awareness campaigns

Select the type(s) of key message(s) relating to infection prevention here below.*Of note, we are mostly interested in the main content of the message, the exact phrasing can be different.*

- [ ] "Keep your vaccinations up to date / get a flu shot"
- [ ] "Hand washing can reduce the spread of infection / Wash your hands"
- [ ] "Don't give antibiotics to your pet; consult a veterinarian first"
- [ ] Other (please specify)

* Did the campaign use any key messages relating to the optimal use of antibiotics in animals?

- ( ) Yes
- ( ) No

Evaluation of antibiotic awareness campaigns

Please specify the key messages used to address the optimal use of antibiotics in animals

Evaluation of antibiotic awareness campaigns

Campaign interventions

* Interventions implemented

- [ ] Distribution or dissemination of communication material
- [ ] Training sessions for prescribers
- [ ] Scientific/professional conferences
- [ ] Publications of articles in medical journals
- [ ] Active promotion / distribution of guidelines
- [ ] Press conferences
- [ ] Public relation activities
- [ ] Activities targeting schools
- [ ] Exhibitions
- [ ] Gimmicks (a method or trick that is used to get people's attention)
- [ ] Other (please specify)

Evaluation of antibiotic awareness campaigns

Which type of printed communication material did you distribute or disseminate?

- [ ] Letters to stakeholders
- [ ] Pamphlets / brochures / leaflets
- [ ] Posters
- [ ] Advertisements in print media
- [ ] Billboards, public transport signs

Where was/were this/these printed material(s) mostly used? (e. g. physicians waiting rooms, hospitals waiting rooms, day care programmes, community hall forums, etc.

* Did you use any online material?

- [ ] None
- [ ] Website
- [ ] Microblog
- [ ] Banners
- [ ] Online documents and pdf
- [ ] Social media channels
- [ ] Videos
- [ ] Advertorials
- [ ] Screen savers
- [ ] If you used any of the above, please specify the number of hits, visits, followers or downloads.

* Did you use television, radio or cinema spots?

- [ ] None
- [ ] Television (public channel)
- [ ] Television (private channel)
- [ ] Radio
- [ ] Cinema

Please specify the number of spots, how often they were broadcasted and when (prime time vs. other)

Evaluation of antibiotic awareness campaigns

Evaluation of the campaign

* Has the impact of the campaign been formally evaluated?

- ◯ Yes
- ◯ No
- ◯ Not sure

Evaluation of antibiotic awareness campaigns

What aspects have been evaluated?

- ☐ Recall of the campaign
- ☐ Antibiotic use
- ☐ Consultation behaviour
- ☐ Antimicrobial resistance rates or sensitivity
- ☐ Unintended consequences (e.g. hospitalizations for complications)
- ☐ Knowledge and attitudes of the public
- ☐ Knowledge and attitudes of healthcare professionals
- ☐ Other (please specify)

* What obstacles did you face while implementing your public awareness campaign? (e.g. lack of funding, lack of political support, etc.)

* Would you recommend a similar type of awareness campaign to a colleague in another country?

- ◯ Yes
- ◯ No
- ◯ Not sure

Evaluation of antibiotic awareness campaigns

If the awareness campaign failed in your country, in your opinion for what reasons did it fail?

Evaluation of antibiotic awareness campaigns

Thank you very much for taking the time to answer this survey.

Your answers will provide highly valuable input to help the planning of future campaigns, that have a key role in the fight against antimicrobial resistance worldwide. If you have any further questions please contact Benedikt Huttner at benedikt.huttner@hcuge.ch or Mirko Saam at m.saam@inscience.ch or +41 78 867 84 83.
